# Clinico-Epidemiological Profile and Outcome of Children with IgA Vasculitis in Aseer Region, Southwestern Saudi Arabia

**DOI:** 10.3390/healthcare9121694

**Published:** 2021-12-07

**Authors:** Samy A. Dawood, Abdoh M. Abodiah, Saleh M. Alqahtani, Ayed A. Shati, Youssef A. Alqahtani, Mohammed A. Alshehri, Syed E. Mahmood

**Affiliations:** 1 Department of Child Health, College of Medicine, King Khalid University, Abha 62529, Saudi Arabia; samyshorbagy8@hotmail.com (S.A.D.); smuadi@kku.edu.sa (S.M.A.); Youssef9811@hotmail.com (Y.A.A.); mohamed8964@hotmail.com (M.A.A.); 2 Pediatric Rheumatology Unit, Department of Pediatrics, Abha Maternity and Children Hospital, Abha 62562, Saudi Arabia; kz-k@hotmail.com; 3 Department of Family and Community Medicine, College of Medicine, King Khalid University, Abha 62529, Saudi Arabia; smahmood@kku.edu.sa

**Keywords:** children, IgA vasculitis, abdominal pain, joints, renal involvement

## Abstract

**Introduction:** Immunoglobulin A (IgA) vasculitis is one of the most common forms of primary vasculitis in children; it typically has a benign course but can be aggressive and require intervention. Aim of the work: The aim of this retrospective study was to evaluate the epidemiological and clinical profile and treatment modalities used for children with IgA vasculitis in the southwestern region of Saudi Arabia. **Material and Methods:** We reviewed the medical records of 89 children admitted to Abha Maternity and Children Hospital in the southwestern region of Saudi Arabia from January 2016 to December 2020 with a confirmed diagnosis of IgA vasculitis according to the European League Against Rheumatism/Paediatric Rheumatology International Trials Organisation/Pediatric Rheumatology European Society criteria. **Results:** Eighty-nine children had a confirmed diagnosis of IgA vasculitis, with 50 boys (56.2%) and 39 girls (43.8%; male-to-female ratio of 1.28:1) and a mean age at diagnosis of 5.87 ± 2.81 years. The mean hospital stay duration was 5.66 ± 4.72 days. Infections preceded 29.2% of the cases, with upper respiratory tract infections comprising 24.7%. Approximately 31.5% of the cases were diagnosed in summer, followed by autumn in 28% of the cases. Rash was present in 100%, arthritis in 72.2%, gastrointestinal tract involvement in 60.7%, and renal involvement in 23.5% of cases. Thrombocytosis and leukocytosis were found in 35% and 46% of all cases, and 52.3% and 47.6.25% of cases with renal involvement, respectively (OR = 2.035, 95% CI: 0.75–5.52 and OR = 1.393, 95% CI: 0.522–1.716, respectively). Approximately 26% of cases experienced relapses. Treatment was conservative in 23.6%, oral prednisolone in 23.6%, and pulse steroid in 45% of cases. Abdominal pain with lower gastrointestinal tract bleeding was the primary indication for initiating pulse steroid treatment. **Conclusions:** There were similarities and differences in the epidemiology and frequency of clinical manifestations of patients with IgA vasculitis compared to previous studies. Children presenting with such epidemiological and clinical profile need to be closely monitored and long-term follow-up is recommended to improve the outcomes.

## 1. Introduction

Immunoglobulin A (IgA) vasculitis involves the deposition of immune complexes containing IgA into small vessels, commonly affecting the gastrointestinal tract (GIT), joints, and kidneys, and less commonly the testicles and central nervous system. IgA vasculitis is predominantly a disease of childhood, occurring most frequently between 3 and 15 years of age, with 50% of all cases occurring in children under 5 years of age. It is more common in males than females, with a male-to-female ratio of 1.5:1, and the peak disease incidence occurs in winter and spring, though there is variability and clusters of cases in other seasons [1,2]. The annual incidence varies by region and can range from 6 to 24 per 100,000 [3].

The etiology of IgA vasculitis is unknown. However, IgA plays a crucial role in the immune pathogenesis of IgA vasculitis, as indicated by the increased serum IgA concentration and IgA deposition in the vessel walls and renal glomeruli seen in patients with IgA vasculitis [4].

IgA vasculitis is primarily diagnosed clinically, as there are no established laboratory diagnostic tests.

The European League Against Rheumatism/Paediatric Rheumatology International Trials Organisation/Paediatric Rheumatology European Society (EULAR/PRINTO/PRES) criteria for IgA vasculitis were used to identify all cases included in this study.

According to this criteria, a patient is classified as having IgA vasculitis (formerly called Henoch–Schonlein purpura) when purpura or petechiae (mandatory) with lower limb predominance is present and accompanied by at least one of the following: (1) diffuse abdominal pain; (2) histopathological findings of leukocytoclastic vasculitis with predominant IgA deposits or proliferative glomerulonephritis with predominant IgA deposits; (3) arthritis or arthralgia; (4) renal involvement (proteinuria: >3 g/24 h or >30 mmol/mg of urine albumin-to-creatinine ratio in a spot morning sample and/or hematuria with red blood cell casts: >5 red cells per high power field or ≥2+ on dipstick or red blood cell casts in the urinary sediment) [5,6].

IgA vasculitis, which has replaced the term Henoch–Schonlein purpura as the nomenclature for this disease, is defined as vasculitis with IgA-dominant immune deposits affecting small vessels [7].

IgA vasculitis is typically a mild and self-limiting disease, and life-threatening complications are rare and may involve the GIT and renal system; long-term complications are associated with renal involvement [8]. The aim of this retrospective study was to characterize and identify the associated clinical, epidemiological and outcome of IgA vasculitis in all hospitalized children aged up to 12 years from January 2016 to December 2020.

## 2. Patients and Methods

### 2.1. Study Design and Patient Selection

This retrospective study included all hospitalized children in the past 5 years with a confirmed diagnosis of IgA vasculitis. We reviewed the medical records of children admitted to Abha Maternity and Children Hospital in the southwestern region of Saudi Arabia in the past 5 years with a confirmed diagnosis of IgA vasculitis according to the European League Against Rheumatism/Paediatric Rheumatology International Trials Organisation/Pediatric Rheumatology European Society criteria.

The study was approved by the Ethical Committee of Scientific Research, King Khalid University. The children enrolled in this study were ≤12 years old and were followed in the pediatric rheumatology clinic for variable periods, with a minimum of 2 years. IgA vasculitis was diagnosed in all cases according to the EULAR/PRINTO/PRES criteria [5,6].

Skin rash was defined as non-thrombocytopenic palpable purpura and/or petechiae symmetrically distributed over the extensor surfaces of the lower legs, buttocks, and arms. GIT involvement was defined as colicky periumbilical or epigastric abdominal pain, nausea, bleeding, or intussusception [9,10].

Arthritis was defined as pain, edema, and functional limitation of the joint [11]. Renal involvement was determined by the presence of hematuria, proteinuria, or blood cell cast, and nephritis was defined as hematuria plus one or two of the following symptoms: hypertension, renal insufficiency, or oliguria [12].

We excluded all cases with missing data and questionable diagnoses. We used the EULAR/PRINTO/PRES criteria [5,6] to identify all cases of IgA vasculitis. We collected the following data for all cases enrolled in the study: age, gender, etiological factors, clinical presentation, complications, type of management, and laboratory workup, including complete blood count, serum urea, creatinine, and electrolyte levels. The patients were followed by the pediatric rheumatology outpatient clinic for a minimum of 2 years, and some cases were followed for more than 3 years.

Thrombocytosis was defined by a platelet count ≥ 500 × 10^9^/L and leukocytosis was defined by white blood cells (WBCs) ≥ 11.0 × 10^9^/L. We defined upper respiratory tract infections (URTIs) as the presence of the common cold, otitis media, pharyngitis, tonsillitis, rhinosinusitis, or laryngitis [13].

### 2.2. Statistical Analysis

SPSS software version 22 (IBM, Armonk, NY, USA) was used to analyze the data, which were presented as frequencies. For inferential statistics, different statistical tests including Pearson’s chi-squared test, and *t* test at 5.00% level of significance were applied to find out the significance differences and correlations assuming normal distribution. SPSS Kolmogorov–Smirnov Test for Normality was also applied.

## 3. Results

The current study included 89 children admitted to the hospital with the diagnosis of IgA vasculitis, consisting of 50 boys (56.2%) and 39 girls (43.8%). Evaluation of family history showed three patients with a family history of bronchial asthma, two patients with a family history of rheumatoid arthritis and Behcet’s disease (father), and two patients with a family history of vesicoureteral reflux. The mean duration of illness before hospital admission was 5.66 ± 4.7 days, and the mean duration of illness before hospital discharge was 10.47 ± 5.79 days.

**Figure 1:**
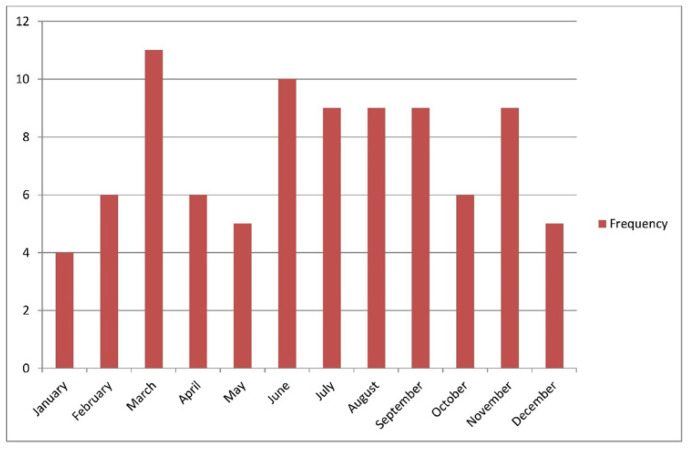
Showing the frequency of IgA vasculitis cases throughout the year.

**Figure 2:**
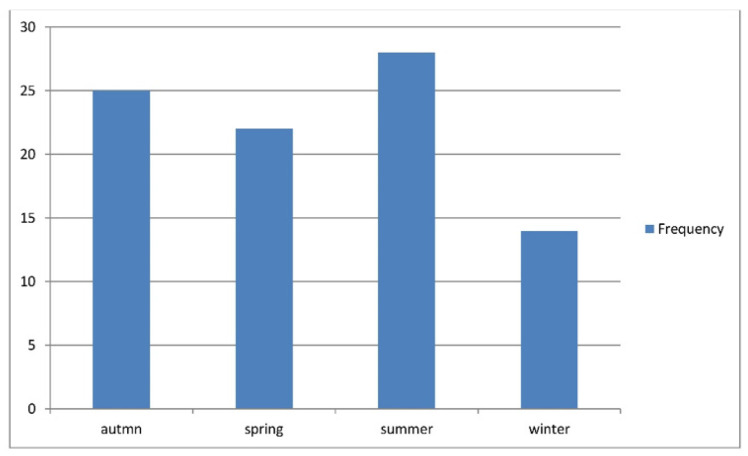
Showing the distribution of IgA vasculitis cases, according to the seasonal occurrence.

Patients’ ages ranged from 1 to 12 years, with a mean of 5.87 ± 2.81 years; 60% of patients were under 7 years of age, with a male-to-female ratio of 1.28:1. Infections preceded the illness in 29.2% of the cases; URTIs comprised 24.7% and gastroenteritis accounted for 4.5%, and these patients had a mean duration of 3.77 ± 2.87 days before the onset of IgA vasculitis and no reported other triggers. The highest percentage of cases occurred in summer (28/89; 31.5%), followed by autumn (25/89; 28.1%; Figure 1 and Figure 2 and Table 1).

Arthritis was the presenting symptom in 66 (74.2%) cases, with a mean duration of 3.69 ± 3.81 days. The mean duration of arthritis was 10.61 ± 5.83 days for patients with arthritis and 10.00 ± 6.00 for patients without arthritis, which was not a significant difference (*p* = 0.662).

The distribution of involved joints included the ankle joint in 50.77% of cases, the knee joint in 12.3% of cases, and both the ankle and knee joints in 29.3% of cases; other joints, such as the elbow, hip, and wrist, were involved to a lesser degree.

**Figure 3:**
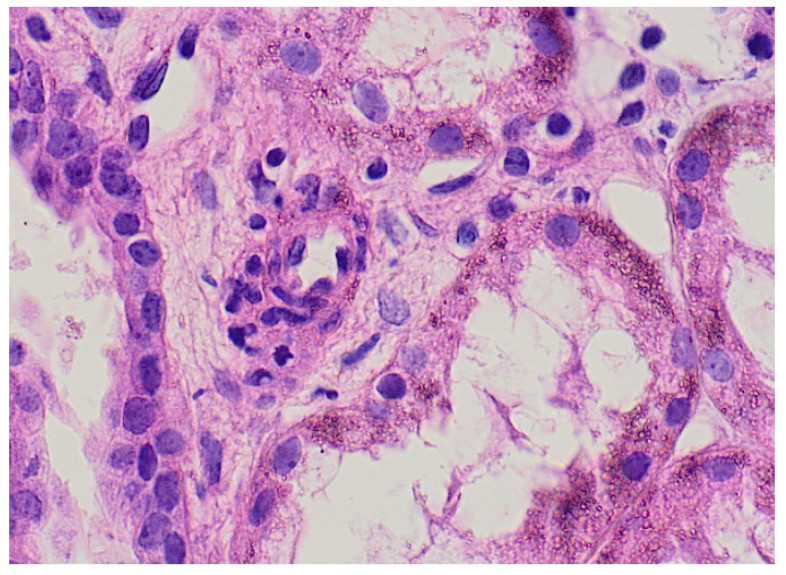
Skin biopsy from 3-year-old boy with picture of leukocytoclastic vasculitis.

The characteristic rash was the most common presentation; it was present in 100% of cases, was primarily distributed over the legs with some inclusion of the buttocks and upper extremities and had a mean duration of 5.25 ± 0.9 days. The rash was bullous and hemorrhagic in two cases, with an excellent response to pulse steroid treatment. Skin biopsy was performed in two patients with findings of leukocytoclastic vasculitis with IgA deposition (Figure 3).

GIT involvement, primarily abdominal pain, was present in 60.7% of cases, with a mean duration of 3.46 ± 5.07 days. GIT involvement consisted of upper and lower GIT bleeding in 29.6% and 3.7% of cases, respectively. Abdominal pain was associated with vomiting and diarrhea in 33.3% of cases. Five patients presented with intussusception, and two of them were treated surgically.

Renal involvement occurred in 23.6% (21/89) of the cases, and microscopic and macroscopic hematuria was present in all cases with renal involvement. There was not a significant difference between the mean duration of illness of 11.285 ± 6.898 days for cases with renal involvement and the 10.22 ± 5.685 days for cases without renal involvement (*p* = 0.464).

**Figure 4:**
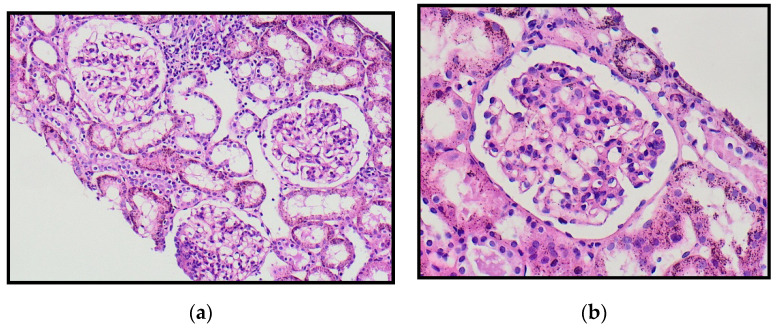
Renal biopsy from a 4-year-old boy with IgA vasculitis with renal impairment, the glomeruli showing mesangial proliferation with no crescent formation. (**a**) Low power and (**b**) high power.

Three cases (14.3%) with renal involvement developed nephritis and required renal biopsy due to persistent hypertension and renal impairment (Figure 4). Proteinuria and WBCs were present in the urine analysis of patients with renal involvement (5/21: 23.8%; Table 2 and Table 3). Leukocytosis was present in 46% (41/89) of all cases and 47.6% of cases with renal involvement. Urinary WBCs were present in 11 patients with renal involvement (11/21; 52.3%).

Scrotal swelling, redness, and pain were the initial presentation in six cases (6.7%), which were followed by the appearance of a rash. Ultrasound with Doppler flow was performed for these cases to rule out torsion and showed scrotal wall edema, increased blood flow in both testes, and epididymis hyperemia, which are consistent with epididymo-orchitis; all cases with testicular involvement responded to steroid and supportive treatment (Table 2).

During laboratory workup, C-reactive protein was positive in 15 cases (15/35; 42.8%), anti-streptolysin O was elevated in 13 cases (13/37; 35.13%), erythrocyte sedimentation rate was elevated in 35 cases (35/74; 47%), and antinuclear antibody was elevated in three cases (3/4; 75%).

Relapse occurred in 22 cases (24.7%), with 11 cases relapsing during the illness (an average of 10 days into the illness) and 11 cases relapsing 1 to 36 months after complete recovery. One case experienced two relapses and three cases experienced three relapses. Skin rash and abdominal pain were the primary manifestations in relapsed patients.

The mean age of relapsed cases was 7.13 ± 2.67 years, while the mean age of non-relapsed cases was 5.61 ± 2.789 years. Nine males (60%) and six females (40%) made up the relapsed cases. Most relapsed cases were diagnosed in autumn and spring. Two out of 15 relapsed cases were preceded by URTI and 80% of relapsed cases received steroids. The duration of treatment for relapsed cases treated with steroids was 18.2 ± 12.167 days, while cases without relapse treated with steroids had a duration of treatment of 14.28 ± 11.685 days (*p* = 0.622).

**Figure 5:**
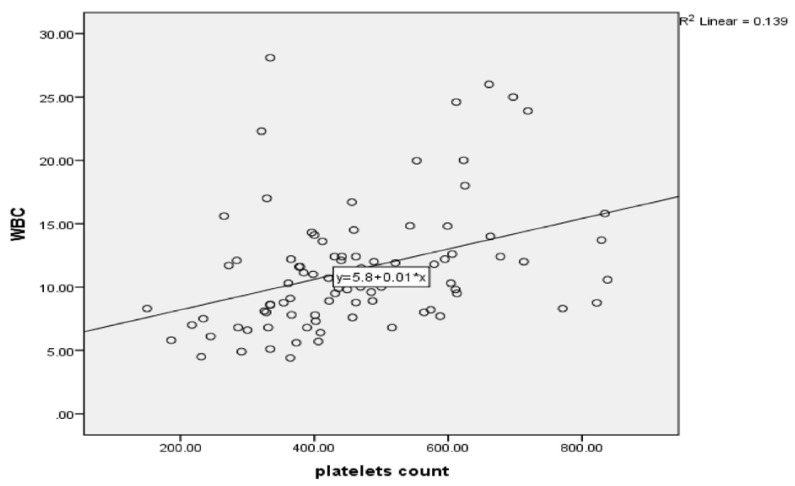
Showing the correlation between WBC and platelets count in IgA vasculitis cases.

Approximately 35% of cases had a platelet count ≥ 500 × 10^9^/L, and 8% had a platelet count above 700 × 10^9^/L. Platelet counts ranged from 150 × 10^9^/L to 883 × 10^9^/L, with a mean of 461.60 × 10^9^/L ± 154 × 10^9^/L. Leukocytosis was found in 46% (41/89) of cases, with a positive correlation between elevated WBCs and platelets (r = 0.373, *p* = 0.001; Figure 5). Of the patients with renal involvement, 52.3% (11/21) had thrombocytosis and leukocytosis (OR = 2.035, 95% CI: 0.75–5.52 and OR = 1.397, 95% CI: 0.522–3.716, respectively). Mean red blood cells were 5.12 ± 5. 57 × 10^12^/L, mean WBCs were 11.35 ± 4.97 × 10^9^/L, and mean hemoglobin was 13.01 ± 1.84 g/L (Table 3 and Figure 5).

Conservative treatment, including adequate hydration, analgesics, bed rest, and non-steroidal anti-inflammatory drugs, were prescribed for 28 patients (31.5%). Oral prednisolone in a dose of 1–2 mg/kg was prescribed for 21 cases (23.6%) for 2 weeks before being tapered for 2 weeks.

**Figure 6:**
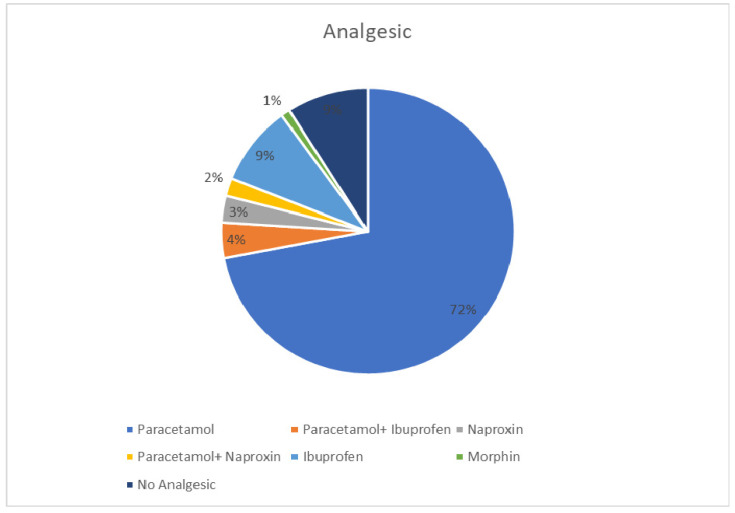
Usage of Analgesics.

Pulse methylprednisolone infusion in a dose of 10–20 mg/kg was prescribed for 40 cases (44.9%), and all cases who received pulse steroids responded after their second or third doses. Pulse steroid treatment was followed by 1 mg/kg oral prednisolone with gradual tapering over 4 weeks. The primary indications for initiating pulse steroids were lower GIT bleeding and severe abdominal pain in 65% of cases, scrotal swelling and pain in 10% of cases, upper GIT bleeding in 7.5% of cases, nephritis in 7.5% of cases, scrotal swelling combined with lower GIT bleeding in 5% of cases, and severe bullous hemorrhagic skin lesions in 5% of cases. We have compared the duration of illness with usage of steroids, and we have observed the significance differences (*p* = 0.00001) (Table 4). As per Figure 6, 72.0% of the patients used paracetamol.

## 4. Discussion

Data were gathered from the medical files of 89 children aged up to 12 years with IgA vasculitis in the past 5 years. All patients were followed in the outpatient clinic for a minimum of 2 years. IgA vasculitis is one of the most common forms of primary vasculitis admitted to our hospital. There were some similarities between the epidemiological and clinical findings observed in this study and those reported in other regions in Saudi Arabia and worldwide [1,14,15,16].

The mean patient age was 5.87 ± 2.81 years and ranged from 1 to 12 years, with males comprising 56.2% and females comprising 43.8% of the sample (male-to-female ratio, 1.28:1). These characteristics are similar to other studies in Saudi Arabia and worldwide, though the mean age in this study was less than other studies due to our inclusion of subjects 12 years old and below [1,14,16,17] Our gender ratio is not in agreement with the results from the study by Al Harbe [18] conducted in the same region of Saudi Arabia, which reported a 1.1:1 female-to-male ratio. In childhood-onset disease, 90% of cases occur under the age of 10 years. It is extremely rare in infants. In children, it has a slight male predominance (1.5:1 male: female ratio) and a decreasing incidence according to increasing age [19].

The cases in our study occurred throughout the year, but the highest incidence occurred in summer (28%), followed by autumn (25%), spring (22%), and winter (14%). This differs from other studies, in which winter and spring were the most common seasons. These findings may relate to the low summer temperatures in our area (22 °C–25 °C) or other reasons that require further research [1,14,16].

Infections preceded the illness in 29.2% of the cases, with URTIs making up 24.7% and gastroenteritis making up 4.5%. These findings are lower than those obtained in studies from other areas in Saudi Arabia [1,14] as well as other countries [20,21,22,23].

Maculopapular rash was reported in 100% of the cases, consistent with studies performed both inside and outside Saudi Arabia [1,14,24].

Arthritis has been reported as the second most common clinical feature of IgA vasculitis in most published studies. In the current study, arthritis was reported in 74.2% of patients, with a mean duration of 3.69 ± 3.81 days. The involved joints in cases with arthritis were primarily the ankle (55.77%), followed by the knee (12.3%) and both the ankle and knee (29.3%); other joints were involved but with lower frequency. The percentages of joint involvement in patients with IgA vasculitis have been variably reported in previous studies, ranging from 53% to 93% [24,25].

GIT involvement, including abdominal pain, upper or lower GIT bleeding, and intussusception, were reported in considerable percentages in studies in Saudi Arabia and other countries [14,26,27,28], with an average incidence of 70%. In this study, 60.7% of cases had abdominal pain, which was associated with upper GIT bleeding in 29.6%, lower GIT bleeding in 3.7%, and vomiting with diarrhea in 33.2%. Intussusception developed in five cases, and three were treated conservatively.

Renal involvement in IgA vasculitis includes transient hematuria, proteinuria, hypertension, and nephrotic syndrome. In most cases, renal involvement is mild and benign, but some cases may progress to renal failure. The percentage of renal involvement is variable, ranging from 20% to 50% [1,23,29,30] In the current study, 23.6% of patients had renal involvement at the time of presentation; all of them had microscopic hematuria, 23.8% had proteinuria, and three had nephritis (3/21; 14.2%). We observed that 47.6% of cases with renal involvement had leukocytosis and 52.25% had thrombocytosis, which are similar findings to those obtained in other studies [12,31].

Approximately 81% of cases with renal involvement were under 8 years of age at the time of diagnosis, while other studies reported older ages, typically those over 10 years of age. This could be due to our sample’s age of 12 years and under. None of the patients developed nephrotic syndrome and three required renal biopsies [32] A retrospective cohort analysis in Taiwan demonstrated that the risk of renal involvement appeared to increase continuously with onset age [33].

Relapse is a common complication of IgA vasculitis, though the percentage varies by study, ranging from 12.4% to more than 50% [31]. In this study, 24.7% experienced a relapse, with 12.3% occurring during or in the first few months after recovery, the rash and abdominal pain being the main clinical manifestations; these results are higher than those reported in other studies in Saudi Arabia [15].

Thrombocytosis and leukocytosis have been associated with IgA vasculitis in children with variable frequencies; thrombocytosis and leukocytosis were reported in 15.3% and 30.7% of cases, respectively, in one Saudi Arabian study. Other studies have reported thrombocytosis and leukocytosis in 44.64% and 53.85% of cases, respectively [1,31,34], while in this study, we found thrombocytosis in 35% and leukocytosis in 46% of cases.

In this study, we used oral prednisolone in 21 cases (21/89; 23.6%), pulse steroid treatment (methylprednisolone 10–20 mg/kg) for an average of 3 days followed by oral prednisolone in 44 cases (44/89; 44.9%), and supportive treatment in 28 cases (28/89; 31.5%).

There is conflicting evidence regarding the role of steroids in IgA vasculitis treatment, as no randomized controlled trials have shown the benefits of steroids against supportive treatment, and some studies have shown limited benefits of steroids [31,34,35] while others have reported that steroids reduce IgA vasculitis symptoms of abdominal pain and arthritis [36,37]. The evidence is also unclear regarding the role of early steroid prescription in preventing future occurrence of nephritis [37,38,39].

Lower GIT bleeding and abdominal pain were the most common reasons for initiating pulse steroid therapy in 65% (26/40) of cases; other indications were scrotal swelling and pain in 10%, upper GIT bleeding in 7.5%, nephritis in 7.5%, scrotal swelling combined with lower GIT bleeding in 5%, and severe bullous skin lesions in 5% of patients.

We used pulse steroids in many cases with the above indications with positive results in decreasing IgA vasculitis symptoms. The use of methylprednisolone in pulse therapy to treat children with IgA vasculitis is not widely reported on, as most previous IgA vasculitis studies are case reports [40,41,42]. A large multicenter observational study of clinical outcomes in hospitalized children with new-onset HSP revealed that early corticosteroid exposure is associated with statistically significant decreased hazard ratios for surgery, endoscopy, and imaging [43]. More studies are required to confirm that corticosteroids are beneficial in the inpatient setting.

### Limitations

A potential limitation of this study is its retrospective nature, so it is possible that some information has been omitted, along with the presence of mis-classification or information bias, and conclusion on causal relationships is difficult to assess. The status at the last documented follow-up was used from the medical records available in this retrospective study. Therefore, some details regarding the cohort of relapsing patients are not available in the information obtained in the data available for medical record review. Further prospective studies are required to minimize such limitations and establish evidence-based practice guidelines.

## 5. Conclusions

In this retrospective study, there were similarities and differences in the epidemiology and frequency of clinical manifestations of patients with IgA vasculitis compared to other studies conducted in Saudi Arabia and other countries. Children presenting with such epidemiological and clinical profile need to be closely monitored and long-term follow-up is recommended to improve the outcomes.

## Figures and Tables

**Table 1:** Epidemiologic characteristics in children with IgA vasculitis.

Characteristics		
**Age in Years**		
Range	1–12	
Mean	5.87 ± 2.812	
Age	No	%
≤6	53	59.56
>6 to <10	29	32.59
>10	7	7.85
**Gender**	No	%
Male	50	56.2
Female	39	43.8
Male to female ratio	1.28:1	
**Seasonal distribution**	No	%
Autumn	25	28.1
Spring	22	24.7
Summer	28	31.5
Winter	14	15.7
**Illness before appearance of disease**	No	%
No illness	63	70.8
URTI	22	24.7
Gastroenteritis	4	4.5
**Mean duration of admission in days**	4.88 ± 2.767	
**Mean duration of illness before admission**	5.66 ± 4.722	

**Table 2:** Clinical features in 89 children with IgA vasculitis.

Skin Rash	*N*	%
Skin rash	89	100
**Arthritis**		
arthritis	66	74.2
Without arthritis	23	25.8
**Joints involved**		
Ankle	30	45.46
Ankle + Elbow	1	1.5
Ankle E + Wrist	3	4.6
Elbow + Knee	1	1.5
Elbow+ Wrist	3	4.6
Knee	8	12.3
Knee + Ankle	19	29.3
Wrist	1	1.5
**GIT involvement**	**54**	**60.7**
Abdominal pains	54	60.7
Bleeding (lower GIT)	16	29.6
Bleeding (upper)	2	3.7
Vomiting and diarrhea	18	33.3
Intussusception	5	9.2
**Renal involvement**	**21**	**23.6**
Microscopic hematuria	18	85.7
Macroscopic hematuria	3	14.3
WBC in urine	11	52.3
Proteinuria	5	23.8
Hypertension	5	23.8
Nephrotic syndrome	0	0
Nephritis with renal impairment	3	14.2
**Testicular involvement**	6	6.7

**Table 3:** Laboratory findings in 89 children with IgA vasculitis.

Laboratory Findings	Number of Positive Tested Cases	%
Anemia (hemoglobin < 110 g/L)	0 (89)	0
Thrombocytosis (platelets > 500, 10^9^/L)	31 (89)	34.8
Thrombocytosis (platelets > 700, 10^9^/L)	7	7.9
Leukocytosis (WBC > 11, 10^9^/L)	41 (89)	46
Elevated ESR (ESR > 20 mm/h)	35 (74)	47
Positive C-reactive protein	15 (35)	43
Positive ASO titer	13 (27)	48

WBC: white blood cell; ESR: erythrocyte sedimentation rate: ASO: anti streptolysin O.

**Table 4:** Comparison of duration of illness and usage of steroid in both groups.

*t*-Test										
Test Description		Levene’s Test for Equality of Variances		*t*-Test for Equality of Means						
		F	Sig.	t	Df	Sig. (2-Tailed)	Mean Difference	Std. Error Difference	95% Confidence Interval of the Difference	
									Lower	Upper
duration of illness	Equal variances assumed	7.861	0.006	11.698	87	0.000	17.46328	1.49290	14.49598	20.43057
	Equal variances not assumed			12.742	73.377	0.000	17.46328	1.37054	14.73204	20.19452

## Data Availability

The datasets used and analyzed in the current study are available from the corresponding author on reasonable request.

## References

[1] Lardhi A.A. (2012). Henoch-Schonlein purpura in children from the eastern province of Saudi Arabia. Saudi Med. J..

[2] Abushhaiwia A.M., Rhuma N.R., Zletni M.A., Ebtisam S. (2018). SM Gr up Clinico-Epidemological Profile of Research Children with Henoch-Schonlein Purpura at Tripoli Children’s Hospital. SM J. Arthritis.

[3] Chen J.-Y., Mao J.-H. (2014). Henoch-Schönlein purpura nephritis in children: Incidence, pathogenesis and management. World J. Pediatrics.

[4] Trapani S., Micheli A., Grisolia F., Resti M., Chiappini E., Falcini F., De Martino M. (2005). Henoch Schonlein Purpura in Childhood: Epidemiological and Clinical Analysis of 150 Cases Over a 5-year Period and Review of Literature. Semin. Arthritis Rheum..

[5] Ozen S., Pistorio A., Iusan S.M., Bakkaloglu A., Herlin T., Buoncompagni A., Brik R., Paediatric Rheumatology International Trials Organisation (2010). EULAR/PRINTO/PRES criteria for Henoch Schönlein purpura, childhood polyarteritis nodosa, childhood Wegener granulomatosis and childhood Takayasu arteritis: Ankara 2008. Part II Final Classif. Cation Criteria.

[6] Yang Y.-H., Yu H.-H., Chiang B.-L. (2014). The diagnosis and classification of Henoch–Schönlein purpura: An updated review. Autoimmun. Rev..

[7] Kiliç B.D., Demir B.K. (2018). Determination of Risk Factors in Children Diagnosed With Henoch-Schönlein Purpura. Arch. Rheumatol..

[8] Jennette J.C. (2013). Overview of the 2012 revised International Chapel Hill Consensus Conference nomenclature of vasculitides. Clin. Exp. Nephrol..

[9] Kawakami T. (2010). New algorithm (KAWAKAMI algorithm) to diagnose primary cutaneous vasculitis. J. Dermatol..

[10] McCarthy H.J., Tizard E.J. (2009). Clinical practice. Eur. J. Nucl. Med. Mol. Imaging.

[11] Saulsbury F.T. (1999). Henoch-Schönlein Purpura in Children: Report of 100 Patients and Review of the Literature. Medicine.

[12] Chan H., Tang Y.-L., Lv X.-H., Zhang G.-F., Wang M., Yang H.-P., Li Q. (2016). Risk Factors Associated with Renal Involvement in Childhood Henoch-Schönlein Purpura: A Meta-Analysis. PLoS ONE.

[13] Zoorob R., Sidani M.A., Fremont R.D., Kihlberg C. (2012). Antibiotic use in acute upper respiratory tract infections. Am. Fam. Physician.

[14] Alharthi A.A. (2016). Henoch-Schonlein purpura in Saudi Arabia: A retrospective study of 27 children in Taif region. Curr. Pediatric Res..

[15] Bukhari E.M., Al-Sofyani K.A., Muzaffer M.A. (2015). Spectrum of Henoch-Schonlein Purpura in Children: A Single-Center Experience from Western Provence of Saudi Arabia. Open J. Rheumatol. Autoimmune Dis..

[16] Akl K. (2007). Childhood Henoch Schonlein purpura in Middle East countries. Saudi J. Kidney Dis. Transplant..

[17] Haghighat M., Hashemi G. (2002). Henoch-Schonlein Purpura In Children: Our Experience With 165 Cases From Southern Iran. Med. J. Islamic Repub. Iran.

[18] Al Harbi N.N. (1996). Henoch-Schoenlein syndrome in children: Experience from southern part of Saudi Arabia. East Afr. Med. J..

[19] Gardner-Medwin J.M., Doležalová P., Cummins C., Southwood T.R. (2002). Incidence of Henoch-Schonlein purpura, Kawasaki disease, and rare vasculitides in children of different ethnic origins. Lancet.

[20] Narchi H. (2005). Risk of long term renal impairment and duration of follow up recommended for Henoch-Schonlein purpura with normal or minimal urinary findings: A systematic review. Arch. Dis. Child..

[21] Huang X., Wu X., Le W.-B., Hao Y., Wu J., Zeng C., Liu Z., Tang Z. (2018). Renal Prognosis and Related Risk Factors for Henoch-Schönlein Purpura Nephritis: A Chinese Adult Patient Cohort. Sci. Rep..

[22] Wang K., Sun X., Cao Y., Dai L., Sun F., Yu P., Dong L. (2018). Risk factors for renal involvement and severe kidney disease in 2731 Chinese children with Henoch–Schönlein purpura. Medicine.

[23] Jauhola O., Ronkainen J., Koskimies O., Ala-Houhala M., Arikoski P., Hölttä T., Jahnukainen T., Rajantie J., Örmälä T., Turtinen J. (2010). Renal manifestations of Henoch-Schonlein purpura in a 6-month prospective study of 223 children. Arch. Dis. Child..

[24] Gül Ş., Sönmez H.E., Çakmak F., Aysel K., Yavuz S. (2019). The clinical spectrum of Henoch Schönlein purpura in children: A single-center study. Clin. Rheumatol..

[25] Peru H., Söylemezoglu O., Bakkaloğlu S.A., Elmas S., Bozkaya D., Elmaci A.M., Kara F., Buyan N., Hasanoğlu E. (2008). Henoch Schonlein purpura in childhood: Clinical analysis of 254 cases over a 3-year period. Clin. Rheumatol..

[26] Chen S.-Y., Kong M.-S. (2004). Gastrointestinal manifestations and complications of Henoch-Schönlein purpura. Chang. Gung Med. J..

[27] Gupta V., Aggarwal A., Gupta R., Chowdhury A.C., Agarwal V., Lawrence A., Misra R. (2018). Differences between adult and pediatric onset Henoch-Schonlein purpura from North India. Int. J. Rheum. Dis..

[28] Ekinci R.M.K., Balcı S., Mart O.O., Tumgor G., Yavuz S., Celik H., Dogruel D., Altintas D.U., Yilmaz M. (2018). Is Henoch–Schönlein purpura a susceptibility factor for functional gastrointestinal disorders in children?. Rheumatol. Int..

[29] Mao Y., Yin L., Huang H., Zhou Z., Chen T., Zhou W. (2014). Henoch–Schönlein purpura in 535 Chinese children: Clinical features and risk factors for renal involvement. J. Int. Med. Res..

[30] Pohl M. (2014). Henoch–Schönlein purpura nephritis. Pediatric Nephrol..

[31] Elmas A.T., Tabel Y. (2016). Platelet Counts in Children With Henoch-Schonlein Purpura-Relationship to Renal Involvement. J. Clin. Lab. Anal..

[32] González-Gay M.A., Ortiz-Sanjuán F., Armesto S., Loricera J., González-Vela M.C., Palmou-Fontana N., González-Gay M.A. (2016). Relapses in patients with Henoch–Schönlein purpura. Medicine.

[33] Liao C.-H., Tsai M., Yang Y.-H., Chiang B.-L., Wang L.-C. (2020). Onset age is a risk factor for refractory pediatric IgA vasculitis: A retrospective cohort study. Pediatric Rheumatol..

[34] Hung S.-P., Yang Y.-H., Lin Y.-T., Wang L.-C., Lee J.-H., Chiang B.-L. (2009). Clinical Manifestations and Outcomes of Henoch-Schönlein Purpura: Comparison between Adults and Children. Pediatr. Neonatol..

[35] Bluman J., Goldman R.D. (2014). Child Health Update Henoch-Schönlein purpura in children Limited benefit of corticosteroids. Can. Fam. Physician.

[36] Jauhola O., Ronkainen J., Koskimies O., Ala-Houhala M., Arikoski P., Hölttä T., Jahnukainen T., Rajantie J., Örmälä T., Nuutinen M. (2012). Outcome of Henoch–Schönlein purpura 8 years after treatment with a placebo or prednisone at disease onset. Pediatric Nephrol..

[37] Shin J., Lee J.S. (2010). Treatment of Severe Henoch-Schoenlein Purpura Nephritis in Children. J. Korean Soc. Pediatric Nephrol..

[38] Dudley J., Smith G., Llewelyn-Edwards A., Bayliss K., Pike K., Tizard J., Tuthill D., Millar-Jones L., Bowler I., Williams T. (2013). Randomised, double-blind, placebo-controlled trial to determine whether steroids reduce the incidence and severity of nephropathy in Henoch-Schonlein Purpura (HSP). Arch. Dis. Child..

[39] Chartapisak W., Opastiraku S.L., Willis N.S., Craig J., Hodson E.M. (2008). Prevention and treatment of renal disease in Henoch-Schonlein purpura: A systematic review. Arch. Dis. Child..

[40] Zaffanello M., Brugnara M., Franchini M. (2007). Therapy for Children with Henoch-Schonlein Purpura Nephritis: A Systematic Review. Sci. World J..

[41] Kang H.S., Chung H.S., Kang K.-S., Han K.H. (2015). High-dose methylprednisolone pulse therapy for treatment of refractory intestinal involvement caused by Henoch-Schönlein purpura: A case report. J. Med. Case Rep..

[42] Hou L., Zhang Z., Du Y. (2021). Leflunomide therapy for IgA vasculitis with nephritis in children. BMC Pediatrics.

[43] Weiss P.F., Klink A.J., Localio R., Hall M., Hexem K., Burnham J.M., Keren R., Feudtner C. (2010). Corticosteroids May Improve Clinical Outcomes During Hospitalization for Henoch-Schonlein Purpura. Pediatrics.

